# What makes the unsupervised monocular depth estimation (UMDE) model training better

**DOI:** 10.1038/s41598-022-26613-0

**Published:** 2022-12-20

**Authors:** Xiangtong Wang, Binbin Liang, Menglong Yang, Wei Li

**Affiliations:** School of Aeronautics and Astronautics, Sichuan University, Chengdu, China

**Keywords:** Aerospace engineering, Electrical and electronic engineering

## Abstract

Current computer vision tasks based on deep learning require a huge amount of data with annotations for model training or testing, especially in some dense estimation tasks, such as optical flow segmentation and depth estimation. In practice, manual labeling for dense estimation tasks is very difficult or even impossible, and the scenes of the dataset are often restricted to a small range, which dramatically limits the development of the community. To overcome this deficiency, we propose a synthetic dataset generation method to obtain the expandable dataset without burdensome manual workforce. By this method, we construct a dataset called MineNavi containing video footages from first-perspective-view of the aircraft matched with accurate ground truth for depth estimation in aircraft navigation application. We also provide quantitative experiments to prove that pre-training via our MineNavi dataset can improve the performance of depth estimation model and speed up the convergence of the model on real scene data. Since the synthetic dataset has a similar effect to the real-world dataset in the training process of deep model, we finally conduct the experiments on MineNavi with unsupervised monocular depth estimation (UMDE) deep learning models to demonstrate the impact of various factors in our dataset such as lighting conditions and motion mode, aiming to explore what makes this kind of models training better.

## Introduction

In recent years, the machine learning based depth estimation methods, which heavily rely on the labeled dataset, have achieved satisfying performance. However, the scarcity of available labeled data, high costs of data acquisition and annotation, limit the quantity and variety of existing deep learning methods. Although the problem of data shortage can be partly solved by unsupervised learning methods with only sparse or even no annotated data, the ground-truth are still needed in experiments for evaluating or testing the generalization performance of the model. Thus, it is still of great significance to obtain a sufficient amount of images with accurate and dense depth information.

The common data acquisition method in real world is not feasible for the depth estimation, especially for aircraft visual navigation because humans cannot manually label a pixel-wise annotation. Building a virtual world to generate synthetic datasets as the intermediate domain with the help of digital simulation technology may be the most feasible way for data generation and labeling at current stage. Since the newly released synthetic datasets^1–4^ are not flexible enough to suit for different needs, e.g, fixed resolution, limited scenes, low data diversity and huge volume, etc, it is difficult to apply them to the dense estimation task in the large scale environment, especially for the depth estimation in aircraft navigation.

**Figure 1:**
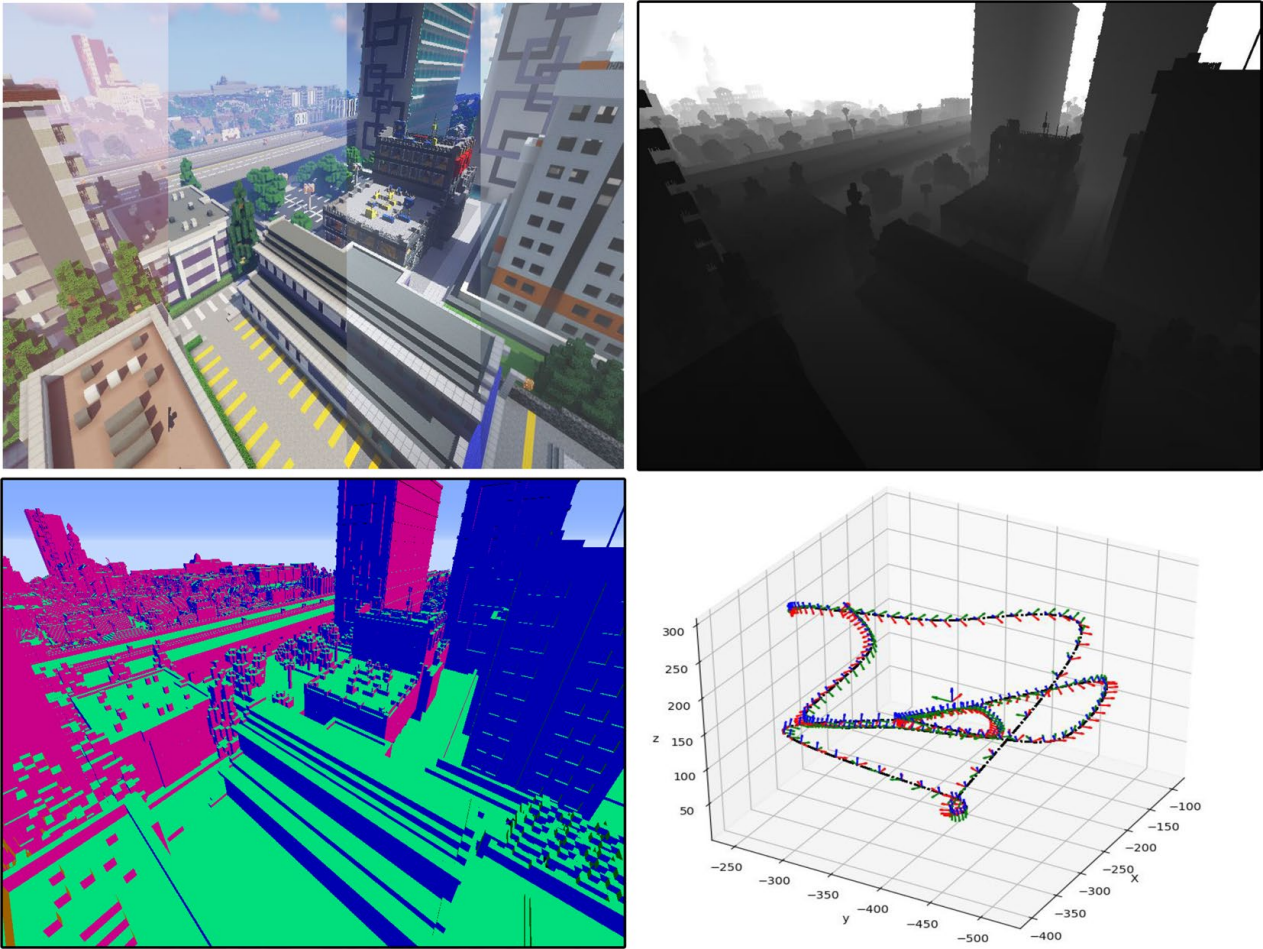
MineNavi dataset provides image sequence, depth map, surface normal map and camera 6 DoF pose in the large scale scene (with over 576 m depth).

Therefore, in this paper, we propose a simple and expandable synthetic dataset generation method, and construct a custom dataset, which is called as MineNavi (Fig. 1). This dataset generation method can not only solve the problem of high cost of real-world data acquisition, but also can narrow the gap between the training domain and the target domain by customizing the synthetic scene that similar with the target domain. Besides, different with conventional studies that adjust models in a fixed dataset to make them close to or superior to the state-of-the-art methods under certain evaluation metrics, we analyze the influences of the changes in datasets on the models. It is very significant because it can not only verify the generalization capabilities of the models to the environment, but also give guidance to construct real-world datasets. In addition, to explore the impact of the various dataset factors on depth estimation models, our constructed MineNavi dataset contains the dense depth maps and surface normal vectors of objects. It will help us to observe the performance of depth estimation model under different factors of the dataset, such as the ego-motion camera, lighting and motion patterns, etc. Our experiments show that these variations on training sets may significantly affect the performance of the models. Finally, unlike the KITTI dataset^5^ applied to autonomous driving, our dataset is mainly oriented to the depth estimation of the large-scale scene in aircraft view, which can not only lead to the development of scene 3D reconstruction^6^ but also provide training data and testbeds for autonomous aircraft with scene perception^7^.

Our contributions are as follows: firstly, we propose an open synthetic dataset generation method and construct MineNavi for the large-scale depth estimation applications. Secondly, we design experiments to report the performance of the baseline models pre-trained on the MineNavi dataset, and reveal the influence of various factors in datasets on depth estimation models. MineNavi dataset is available on Kaggle platform^8^.

**Figure 2:**
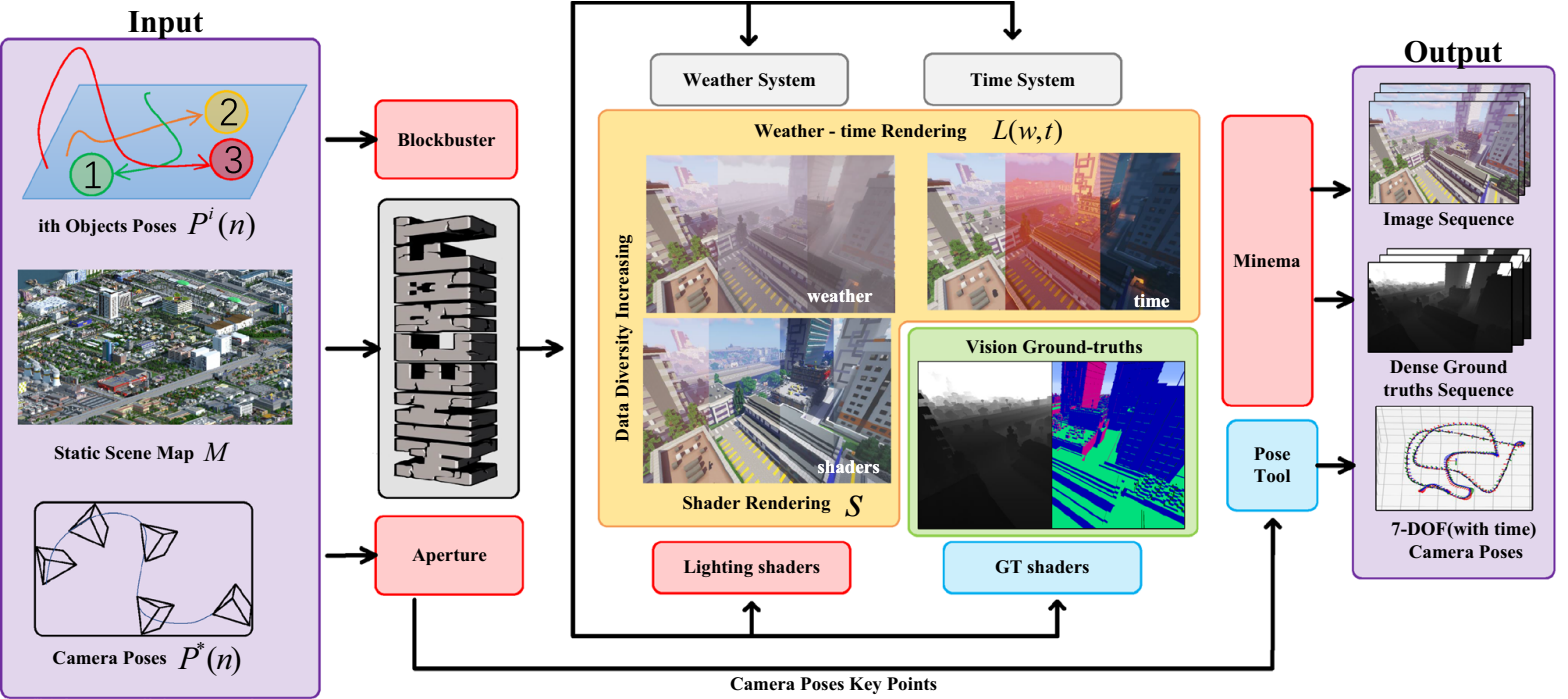
Data generation pipeline. We use some open source tools and tools provided by ourselves to achieve efficient data generation.

## MineNavi: a synthetic dataset of large scale scenes

Using MineCraft to construct a dataset is not a novel idea for computer vision community^9^, we here to utilize it for depth estimation in aircraft visual navigation application. Our data generation method contains four steps: map loading, camera moving path setting, shader and lighting conditions setting, and ground-truth acquisition. Figure 2 shows the pipeline of data generation process.

Not only the environment features, such as the scene structure and lighting condition, affect the performance of depth estimation models, but also the particular dynamical parameters, such as moving targets in the environment and ego-motion of the aircraft, play important roles in benchmark datasets for models’ training and evaluation. Accordingly, each image frame of the dataset can be parameterized as:

1 $$\begin{aligned} I \left[ M,P^{\star }(n), s,L(t,w) \right] \end{aligned}$$

where $P^{\star }(n)$ represents the 6 DoF camera motion paths, *n* is the quantified timestamp of the path, *M* is the map of the scene, *s* is the shader that renders the world, *L*(*t*, *w*) is the lighting condition. *t* is the time in a day, and *w* indicates the weather conditions.

### Scene construction

Although a lot of work^2,10^ build the scenes based on 3D modeling software such as *Blender* and *Maya*, the construction of large-scale 3D scenes is still a relatively time-consuming and laborious task. Besides, the limited scenes diversity will lead to the over-fitting situation of the models. *MineCraft* community^11^ has extremely rich scene maps and users can freely build the required scene to generate specific dataset. Since the aircraft navigation is always involved in the large-scale scenes, the negative effects of the jagged features of objects in MineCraft can be ignored.

In order to increase the diversity of data, we use different shaders and lighting conditions. MineNavi dataset cooperates with the time and weather system according to different light and shadow styles to generate multiple style data.

**Figure 3:**
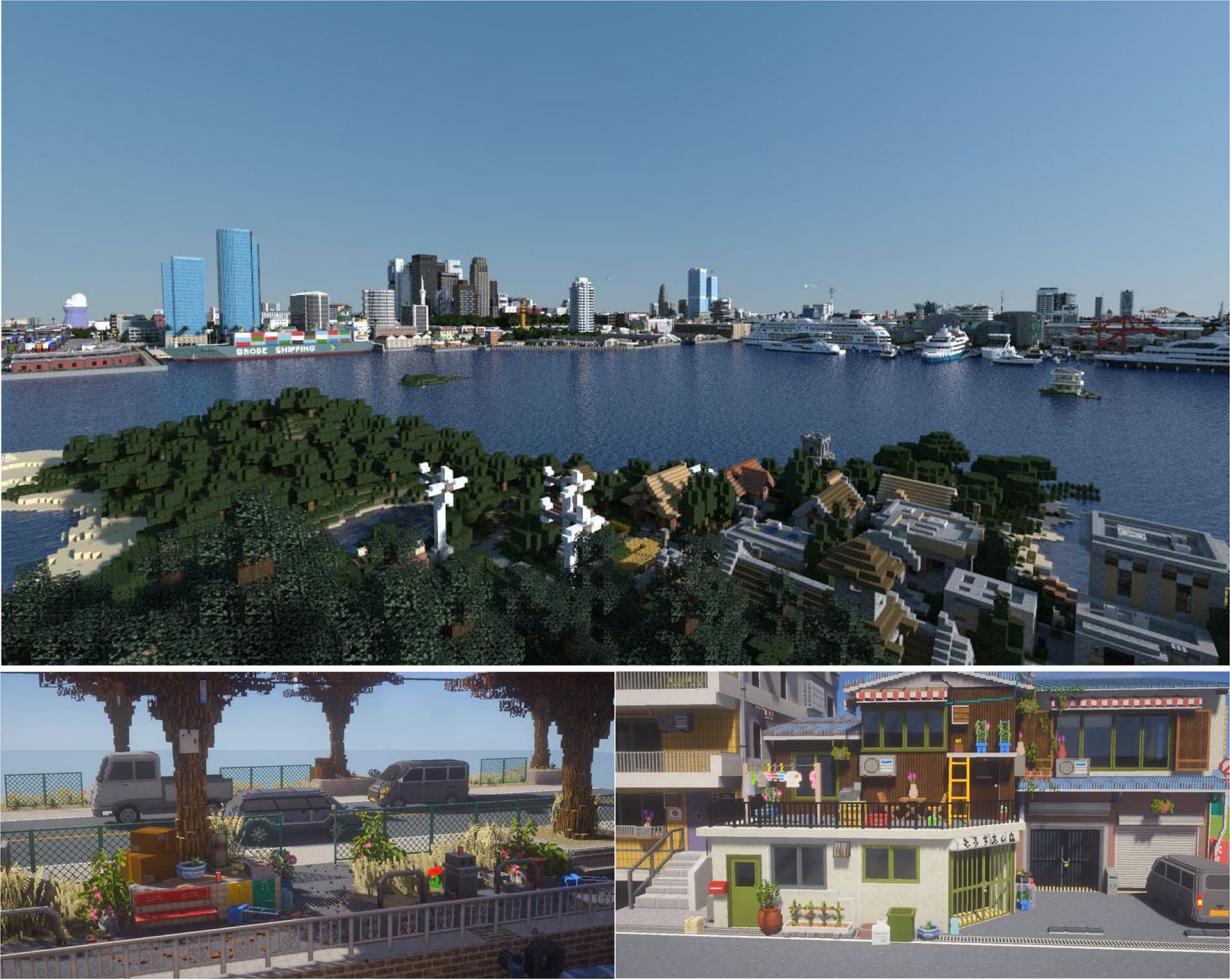
Virtual world constructed in *MineCraft*. Up: The open virtual world *AudiaCity* that we used to build our dataset. Down: Users can achieve higher resolution scenes or buildings by applying plugins that adjust the blocks to small size.

The construction in MineNavi based on the block is very simple and flexible. In order to build a more refined scene, users can use plug-ins to adjust the size of the block to achieve more complex objects (see Fig. 3).

### Camera paths setting

Base on previous study^12–14^, we have found that the unsupervised monocular depth estimation methods are very sensitive to the camera motion in the training.

Therefore, we develop different camera paths and generates corresponding datasets for experiments.

Unlike lighting and other factors that can be quantified as a scalar, a moving camera has 6 continuous degrees of freedom.

Therefore, for a training triplets, we propose a quantitative scalar $\lambda$, i.e., quasi-axis rate to generate datasets of the motion paths according to $\lambda$, and analyze the pros and cons of the data under different $\lambda$. The $\lambda$ can be formulated as:

2 $$\begin{aligned} \lambda (n)= & {} \frac{{\bar{\phi }}(n) {\dot{t}}(n) }{\Vert {\bar{\phi }}(n)\Vert \Vert {\dot{t}}(n)\Vert } \end{aligned}$$

3 $$\begin{aligned} {\bar{\phi }}(n)= & {} \frac{\phi (n+1) + \phi (t-1)}{2} \end{aligned}$$

4 $$\begin{aligned} {\dot{t}}(n)= & {} t(n+1) - t(n-1) \end{aligned}$$

5 $$\begin{aligned} P^\star (n)= & {} (\phi (n),t(n) ) \end{aligned}$$

$\phi (n)$ is the rotation angle of camera visual axis at time *n*, calculated from $R\in SO(3)$,and $t(n) = [x,y,z]$ is the camera position vector at time n. When $\lambda =0$, the camera moves parallel to the visual axis, when $\lambda =1$, the camera moves perpendicular to the visual axis.

**Figure 4:**
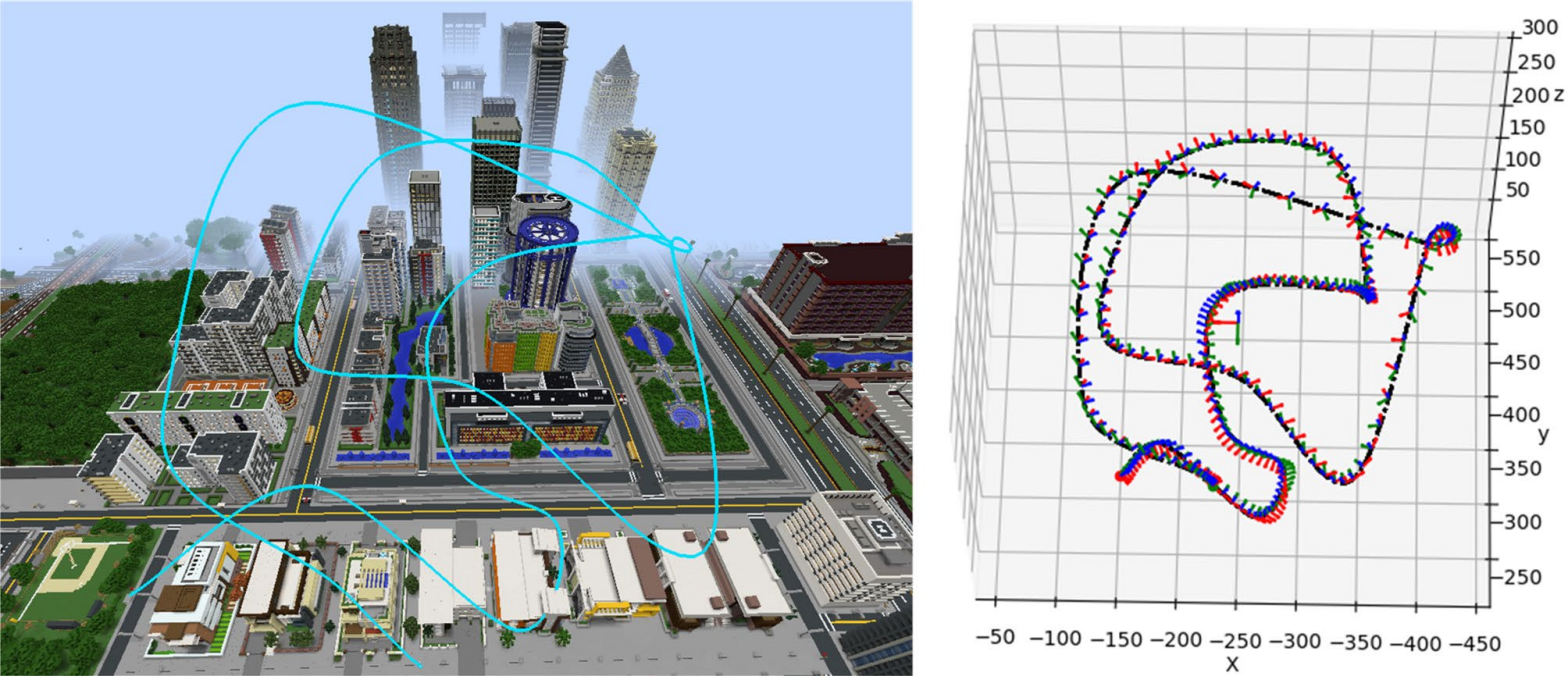
The accurate camera 6 DoF pose at any timestamp in the path is gotten by exporting the key points of the *Aperture* path and interpolating.

In MineNavi, we can set the key points manually or automatically by using *Aperture*^15^ and obtain the full path that matched with image sequence through the interpolation algorithm (see Fig. 4). The generated path has high enough dynamics, and the pose transformation is much larger than the general real-world data captured by UAVs.

### Moving objects in the scene

The dynamic objects in a practical environment may have a great influence on unsupervised monocular depth estimation method. Many previous work^16–18^ focused on how to remove the negative effects of dynamic objects in monocular depth estimation, but due to the very limited dataset, the progress is far from satisfactory. In order to simulate the influence of a moving object in the synthetic dataset on the depth estimation, the construction of the scene containing moving objects can be further parameterized as:

6 $$\begin{aligned} M\left[ P^1(n),P^2(n),\ldots ,P^m(n) \right] \end{aligned}$$

where $P^i$ is the path of the ith moving objects in the scene. Each of the dynamic object can be modeled as custom shape by *Blender* or other 3D software and set their paths by using *BlockBuster*^19^. Note that we have no involved the moving object in our proposed dataset yet due to its negative effects on depth estimation mode.

### Generating ground-truth annotations

The shader can perform color mapping on the 3D information of the scene, which acts as a ground-truth as shown in Fig. 1.

We use the *DepthMap* rendering plug-in to export the corresponding error-free, pixel-level dense depth map that matches the image in sequences. In addition, we provide a surface normal rendering plug-in *SurfMap* to support surface normal estimation tasks.

Thus, the datasets construction method proposed in this study can generate a large number of customized datasets at a very low cost.

### Datasets building

We constructed several datasets by MineNavi, as shown in Table 1. MNv1.0 contains 40 scenes and total of 2000 images (50 images per scene) rendered by sildurs-middle shader with the sunny weather at noon, MNv1.1 and MNv1.0 are identical except for using sildurs-high shader as the renderer. MNv1.2 has raw, sildurs-middle and sildurs-high renders. MN1.3 is the largest dataset we have built, containing 3 blur conditions (low, middle, high), five lighting conditions (morning, noon, afternoon, night, rain), 324 scenes, and a total of 168,200 images. Compared with MNv1, MNv2 differs mainly in the motion patterns and scenes, which are centered around a certain central point with three lambda motions, i.e., $\lambda =1,\sqrt{2}/2, 0$.

**Table 1:** Builded MineNavi datasets.

Dataset	Num. of images	Rendering quality	Num. of scenes	Lighting conditions	Motion blur	$\lambda$
MNv1.0	2000	Middle	40	Noon	–	1
MNv1.1	2000	High	40	Noon	–	1
MNv1.2	9600	All	40	All	–	1
MNv1.3	162,000	High	324	All	All	1
MNv2.0	16,200	High	40	Noon	–	1
MNv2.1	16,200	High	40	Noon	–	$\sqrt{2}/2$
MNv2.2	16,200	High	40	Noon	–	0

## Experiments

In this section, we verify the feasibility and credibility of the MineNavi dataset in the training of the depth estimation model, and explore the impact of dataset varieties on the unsupervised monocular depths estimation model. Thus, we will demonstrate that 1) the depth estimation model can improve generalization through pre-training on MineNavi. 2) it is desirable to exploit the influence of data to model caused by various factors of the dataset. We prepare monodepth2^20^ and its two variants monodepth2-3D and monodepth2-3Ds as the test models on our proposed datasets. We also present using Sequential Heat-map of Photometric-error Histogram (SHPH) to verify whether an image sequence is compatible with depth estimation model training intuitively.

### MDE models

**Figure 5:**
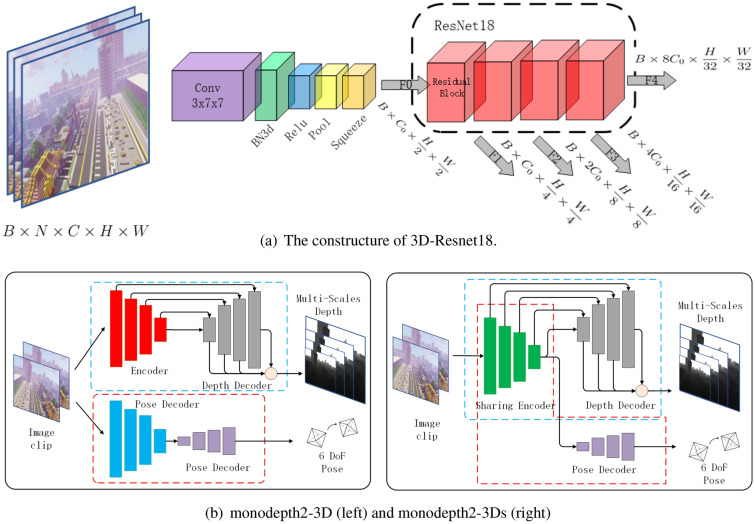
For spatiotemporal feature learning, We build a 3D encoder and apply it into monodepth2 to build monodepth2-3D and monodepth2-3Ds.

Unsupervised depth estimation includes monocular methods^12–14,21–24^ usually contain a single-view depth and a multi-views pose network, to compute the depth. With the similar principle, we use a test model monodepth2^20^ and its variants as baseline, which are shown in Fig. 5.

Inspired by spacial-temporal methods in scene understanding^25,26^, the first variant of monodepth2 is monodepth2-3D, i.e., replace the encoder with a 3D encoder for improving the efficiency of training frames, which can enhance the richness by extracting the temporal features from multiple images^17,27,28^. What’s more, as mentioned by previous work^29^ that if there is structural similarity among candidate tasks, it is reasonable to assign just one encoder to extract identical features and recover required information by task-oriented decoders respectively^20,21^. Thus, we apply the model that using a single encoder to extract the mixed features for depth or pose estimation network as second variants of monodepth2, i.e., monodepth2-3Ds.

### Apply MineNavi to MDE models

**Figure 6:**
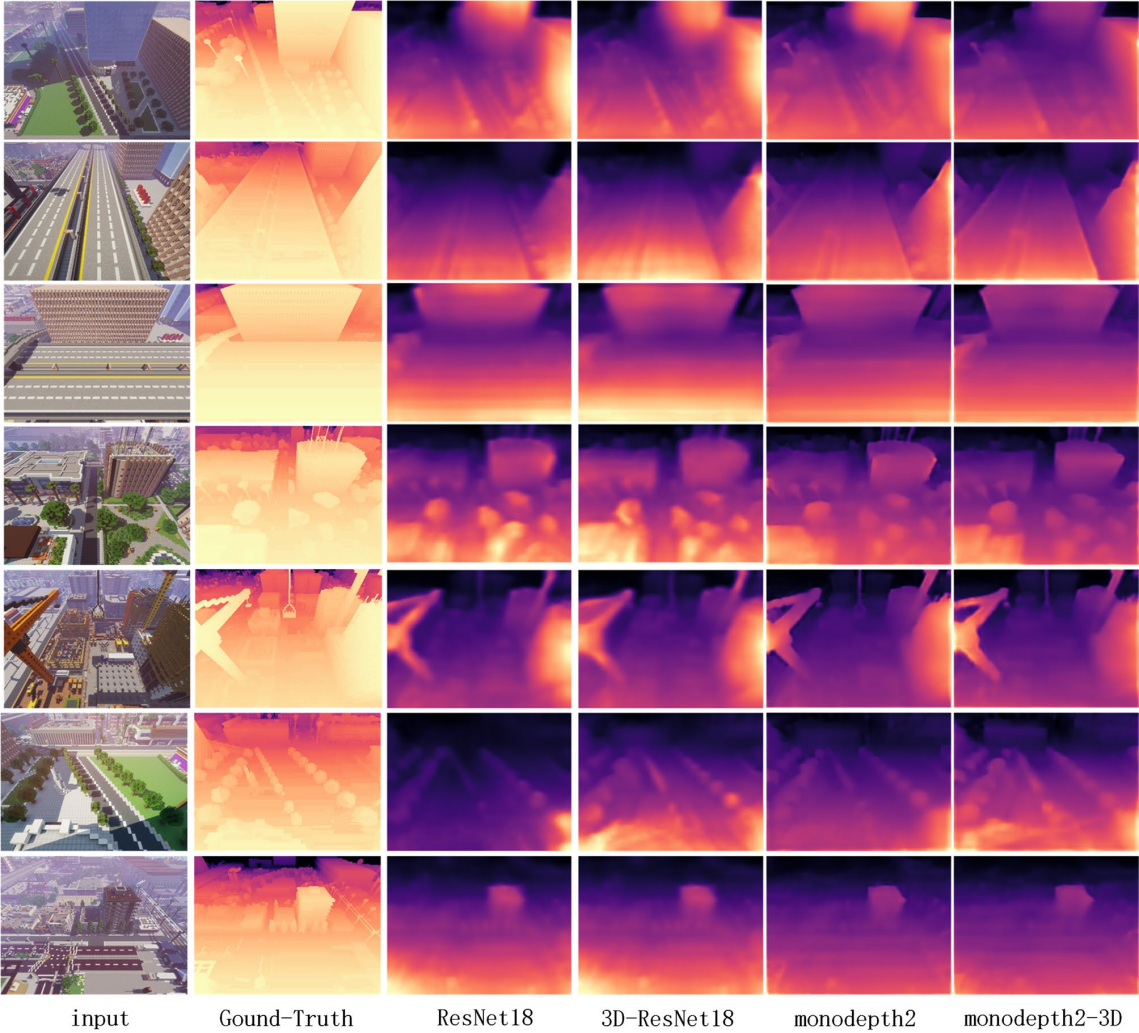
Qualitative results of different models in MineNavi.

We present two variant models by changing their encoder, and apply them into frameworks of supervised training and unsupervised training (monodepth2) on MNv1.0, MNv1.1 and MNv1.2. For comparison, we also prepare the model Table 2 shows the results of models with single-frame and multi-frame input and the models on MN achieve similar or even better results by simply replacing the encoder from ResNet18 to 3D-ResNet18. Obviously, depth information is embedded under the multi-frame image sequence, which can assist the model to recover depth better. The quantitative results are shown in Fig. 6.

**Table 2:** Quantitative results in MN datasets. First six model are trained in supervised and the rest are unsupervised (monodepth2).

Model	Dataset	Error $\downarrow$				Accuracy $\uparrow$		
		AbsRel	SqRel	RMS	RMSlog	$<1.25$	$<1.25^2$	$<1.25^3$
ResNet18	MNv1.0	0.198	1.318	5.679	0.268	0.731	0.923	0.972
3D-ResNet18	MNv1.0	**0**.**194**	** 1.197**	**5**.**453**	** 0.259**	**0**.**749**	**0**.**932**	**0**.**973**
ResNet18	MNv1.1	0.207	1.479	5.832	0.274	0.721	0.920	0.968
3D-ResNet18	MNv1.1	** 0.181**	** 1.159**	**5**.**372**	**0**.**250**	** 0.761**	** 0.935**	**0**.**976**
ResNet18	MNv1.2	0.142	0.707	4.455	0.181	0.846	0.961	0.986
3D-ResNet18	MNv1.2	**0**.**129**	** 0.675**	**4**.**384**	** 0.172**	**0**.**861**	**0**.**966**	**0**.**987**
Monodepth2	MNv1.1	**0**.**212**	**1**.**426**	7.054	0.295	0.706	0.902	0.959
Monodepth2-3D	MNv1.1	0.245	1.833	**5**.**919**	**0**.**273**	**0**.**750**	**0**.**925**	** 0.965**
Monodepth2	MNv1.2	0.170	**0**.**974**	**5**.**782**	0.211	0.798	0.941	0.977
Monodepth2-3D	MNv1.2	0.165	1.170	5.965	0.211	**0**.**800**	**0**.**942**	**0**.**977**
Monodepth2-3Ds	MNv1.2	**0**.**160**	0.991	5.899	**0**.**208**	0.809	0.943	**0**.**977**

The best results in each dataset are shown in bold.

### Generalization of MineNavi

We execute the models pre-training on MineNavi datasets with linear camera moving path. In order to evaluate the influence of data diversity on the performance, we use MNv1.0, MNv1.1 and MNv1.2 for model pre-training. For comparison, we also prepare the models that are trained from scratch and ImageNet^30^. Although the model pre-trained on ImageNet by classification task has structural difference compare with the model that trained on similar target task, it is still the most popular method in depth estimation task.

#### Fine-tune on KITTI

We conduct fine-tuning on the KITTI with monodepth2, monodepth2-3D and monodepth2-3Ds pre-trained from scratch, ImageNet, MNv1.2 and MNv1.3 for 10 epochs. Note that we have removed the mask mechanism and reduced the epochs for simple training without affecting the final conclusion, so the results may be different from the original monodepth2^20^.

From Table 3 it can be seen that the performance of monodepth2 and monodepth2-3D pre-trained on ImageNet is better than that pre-trained on MNv1.2 and scratch, but worse than MNv1.3. The MineNavi has a strong generalization capability compared to the KITTI. As mentioned before, MNv1.2 and MNv1.3 are only different in lighting condition and data volume. Therefore, the diversity of lighting conditions effectively improves the generalization capabilities of the models.

**Figure 7:**
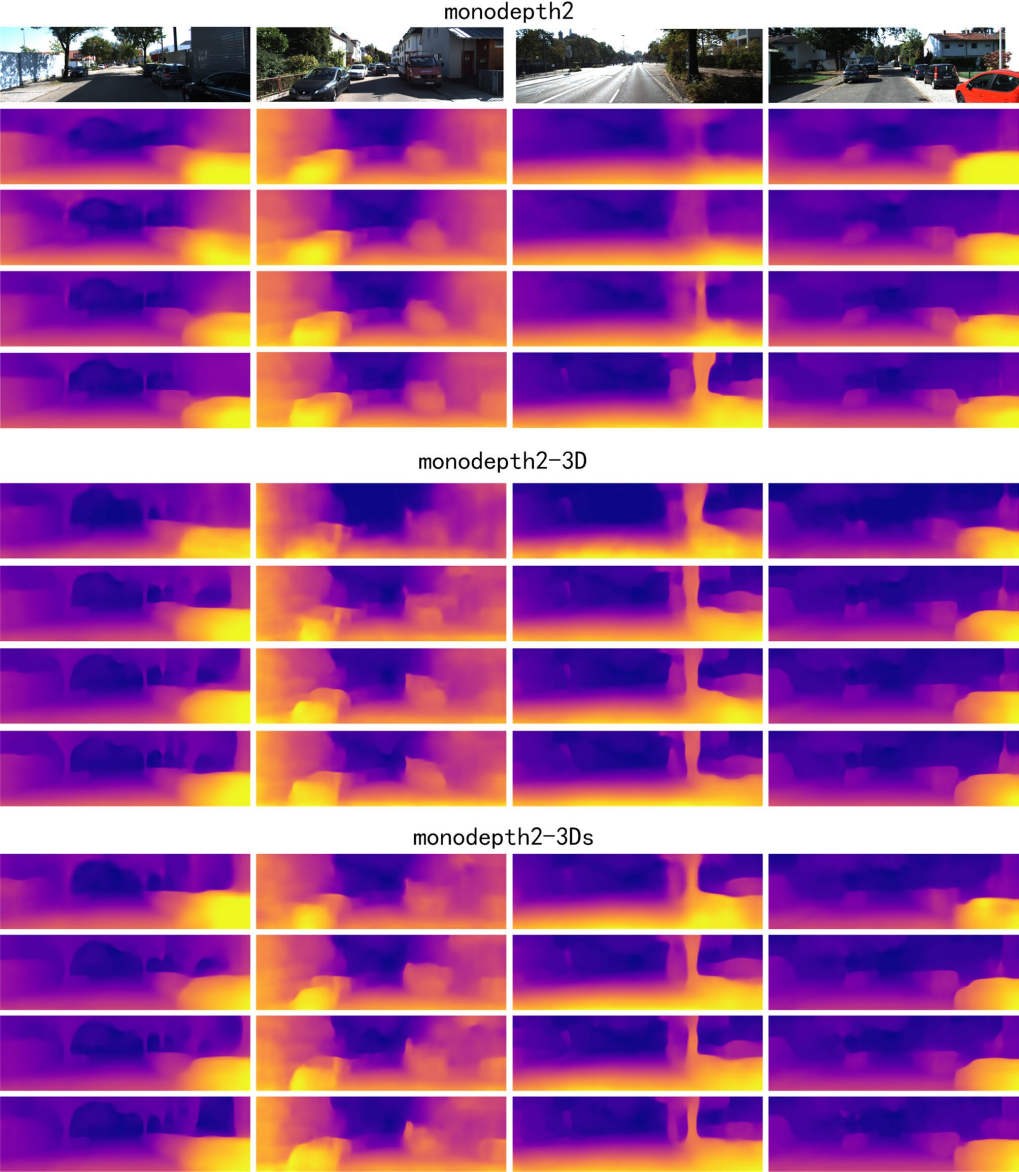
Qualitative results of different models in KITTI.In these models, we used different pre-training weights and a completely consistent tuning process. The results for each model are, from top to bottom, train from scratch, ImageNet, MNv1.2 and MNv1.3.

**Figure 8:**
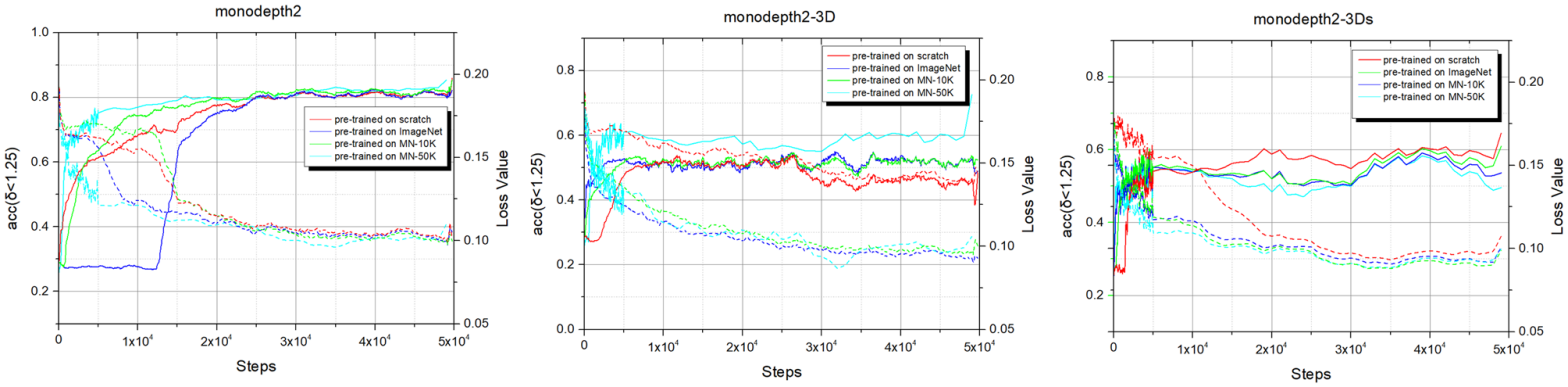
Fine-tuning curves of three test models on KITTI. Solid curves denote accuracy ($\delta \le 1.25$) metric of depth estimation and dash curves denote training loss.

Compared with the other datasets, the model of monodepth2-3Ds pre-trained on ImageNet has the better performance. This is mainly because excessive noises in KITTI, e.g, the moving objects deteriorate the robustness of the network performance of the shared encoder, but the large amount of data of ImageNet can make the model more robust^31^. Note that although MineNavi dataset is much smaller the ImageNet, it has competitive performance with ImageNet in depth estimation model training. The quantitative results are show in Fig. 7 which matches with Table 3. It can be seen that the depth map obtained by the MN-trained model has sharper edges compared to the ImageNet with the trained model, which also indicates that the model can generalize better to similar tasks by unifying the task with the trained model. We also provide fine-tuning curves on Fig. 8 and it shows the value in generalization of our MineNavi dataset.

**Table 3:** Quantity results of various MDE models in KITTI with different pre-trained datasets. The best result are bolded and the second best are underlined. Since we have only 10 epochs of fine-tune and without masking mechanism, the results are different from the original paper of monodepth2^20^.

Models	Pre-trained datasets	Error $\downarrow$				Accuracy $\uparrow$		
		AbsRel	SqRel	RMS	RMSlog	$<1.25$	$<1.25^2$	$<1.25^3$
Monodepth2	Scratch	0.141	1.117	4.797	0.205	0.839	0.948	0.980
	ImageNet	0.135	**1**.**007**	4.668	0.200	0.845	0.950	**0**.**980**
	MNv1.2	0.138	1.095	4.722	0.204	0.843	0.949	0.979
	MNv1.3	**0**.**130**	1.055	**4**.**630**	**0**.**196**	**0**.**856**	**0**.**953**	** 0.980**
Monodepth2-3D	Scratch	0.170	**1**.**453**	5.758	0.247	0.775	0.916	0.963
	ImageNet	0.163	1.857	5.529	0.233	0.807	0.930	0.968
	MNv1.2	0.167	2.468	5.671	0.230	0.820	0.932	0.966
	MNv1.3	**0**.**153**	1.639	** 5.356**	** 0.224**	**0**.**820**	**0**.**936**	**0**.**970**
Monodepth2-3Ds	Scratch	0.161	**1**.**660**	** 5.449**	0.228	0.805	0.930	0.970
	ImageNet	**0**.**158**	2.447	5.511	**0**.**220**	**0**.**839**	**0**.**938**	** 0.970**
	MNv1.2	0.165	2.361	5.535	0.229	0.820	0.934	0.968
	MNv1.3	**0**.**158**	2.133	5.555	0.223	0.829	0.936	0.969

**Figure 9:**
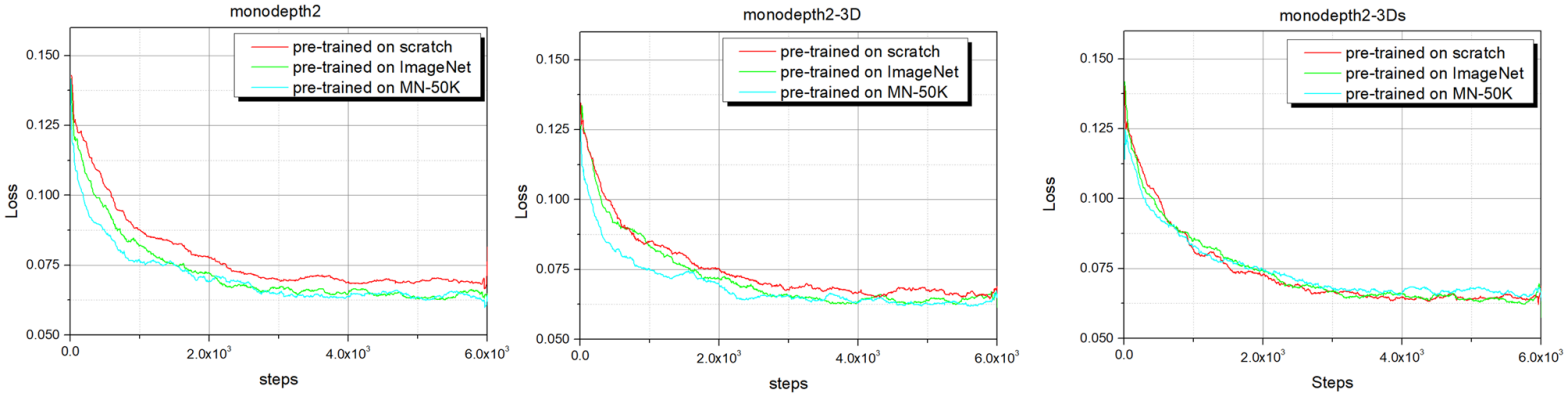
Fine-tuning curves of three test models on FPV.

#### Fine-tune on FPV

Since there is no ground-truth in the FPV dataset, we have to compute the distances between the models in different domains based on the loss value^29^. The closer the migration distance is, the better the pre-training dataset can be generalized to the target domain.

Compared the losses curve among of different pre-trained models that are fine-tuned on FPV in Fig. 9, MineNavi pre-trained models converge faster than the others. The reason behind probably comes from that the MineNavi dataset is closer to the FPV dataset than ImageNet in terms of environment scenes. What’s more, compared with the ImageNet pre-trained model through the task of objects detection, the MineNavi pre-trained model through the task of depth estimation has learned geometric representation^32^ during the pre-training, which leads the model converge faster when the target task has structural similarity^29^ with source task. Note that, with the continuous expansion of the dataset, MineNavi can realize a more satisfied performance.

### Factors that affect the train of MDE

Due to the expandable characteristics of the MineNavi dataset, we can easily generate customized datasets with different variation factors to avoid the over-fitting. It also a helpful way to discover the impacts of factors of datasets on the models. Thus we conduct experiments to explore how the factors in dataset can affect MDE model, including the shader, lighting conditions, motion blur, ego-motion and velocity of training image sequence.

#### Impact of shaders

The MineNavi dataset can generate the rendered image sequences sampled on the same path through different shaders, which allows us to quantitatively evaluate the impacts of the synthetic world design and the quality of other rendering parameters on the algorithm performance. We apply  Sildurs^33^ to adjust the image rendering quality and build three training datasets index *Raw, middle-sildurs* and *high-sildurs* of MNv1.2. All of them are captured in an identical scene with linear camera motion and collected for about 10000 images. The only differences among them are shader setting: *Raw* is rendered by no shader, *middle-sildurs* uses sildurs with middle performance and *high-sildurs* uses high-performance shader. We apply random initial weights encoder to monodepth2 and train it on above three datasets. We use cross-evaluation on each trained model, i.e., evaluate every model on all datasets. The qualitative results are shown in Table 4.

**Table 4:** The performance of the accuracy of the generated dataset under different rendering shaders. Here *AbsRel* and $\delta <1.25^1$ are used as error and accuracy indicators. The best result in each row has been underlined and the optimal result has been bolded.

Train sets (AbsRel $\backslash \delta ^1$)	Test datasets		
	*Raw*	*Middle-sildurs*	*High-sildurs*
*Raw*	0.207$\backslash$ 0.689	0.326$\backslash$0.524	0.311$\backslash$0.528
*Middle-sildurs*	0.436$\backslash$0.425	0.148$\backslash$ 0.813	0.158$\backslash$0.774
*High-sildurs*	0.439$\backslash$0.430	0.156$\backslash$0.778	**0.143**$\backslash$ **0.816**

It shows that as the training scenes rendered gradually improve, the performance of the depth estimation model improves consequently. Besides, compared with a model that is trained on less-texture data and tested on rendered data, the model that is trained on rendered data and tested on less-texture data brings a worse result. It is consistent with the fact that the rendering performance will promote the robust of the model during the training.

#### Lighting conditions

Previous study^20,34^ show that during the depth estimation model training, the low-texture areas caused by insufficient lighting or overexposure will produce problem pixels in depth estimation.

**Figure 10:**
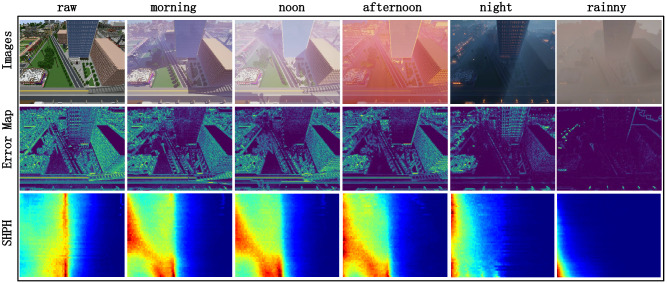
Sequence under different lighting conditions. Photometric error map (row 2) and SHEH map (row 3).

**Figure 11:**
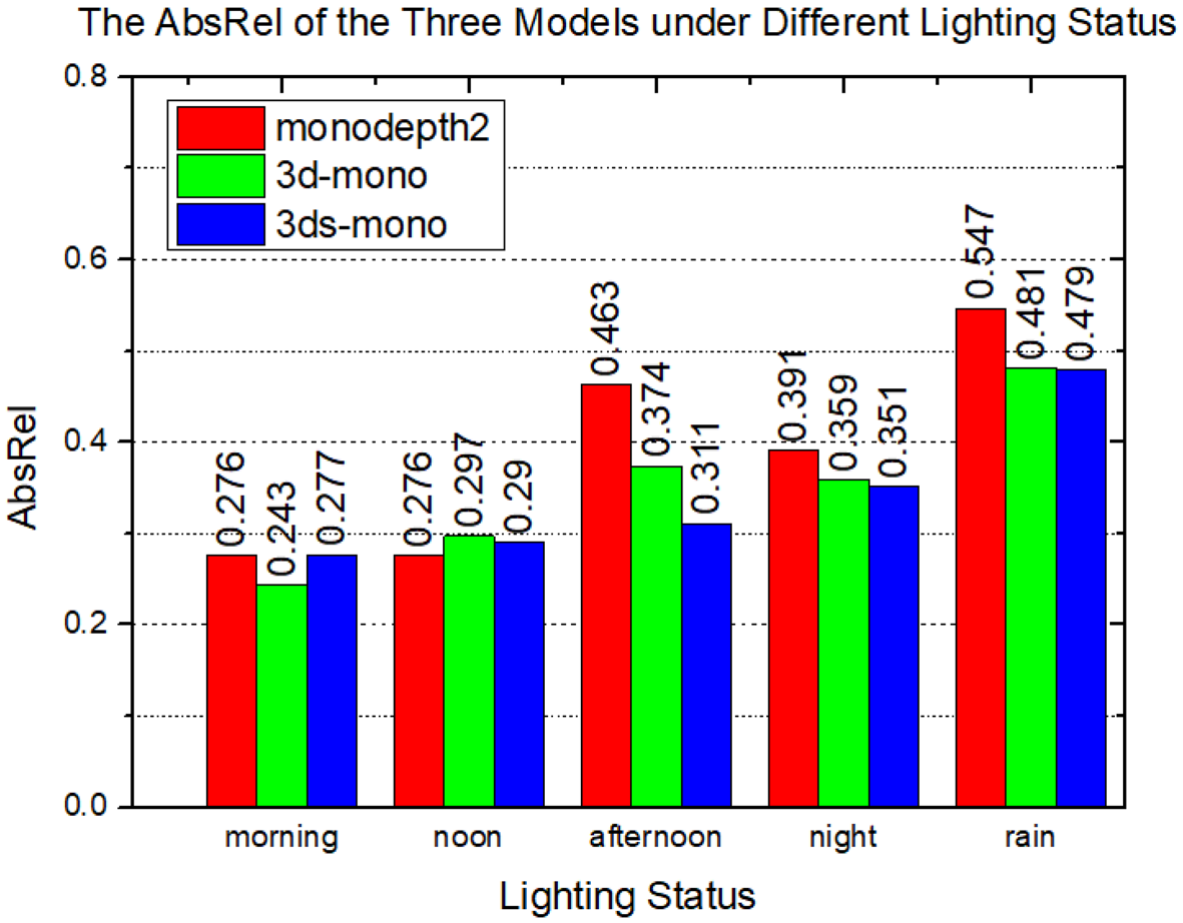
Models trained with datasets that various in lighting conditions show different *AbsRel*.

**Table 5:** Quantity results in MineNavi with different lights.

Models	Dataset index	Error $\downarrow$				Accuracy $\uparrow$		
		AbsRel	SqRel	RMS	RMSlog	$<1.25$	$<1.25^2$	$<1.25^3$
Monodepth2	Morning	0.276	8.491	36.672	0.289	0.694	0.885	0.947
	Noon	0.276	11.284	49.412	0.321	0.665	0.858	0.931
	Afternoon	0.463	26.176	67.480	0.534	0.375	0.647	0.803
	Night	0.391	18.674	57.397	0.432	0.470	0.748	0.875
	Rain	0.547	30.597	54.880	0.520	0.395	0.671	0.818
Monodepth2-3D	Morning	0.243	7.469	36.867	0.263	0.737	0.903	0.957
	Noon	0.297	11.108	46.373	0.324	0.622	0.852	0.936
	Afternoon	0.374	13.967	45.221	0.391	0.478	0.779	0.910
	Night	0.359	15.065	49.514	0.392	0.490	0.780	0.907
	Rain	0.481	24.776	57.464	0.492	0.386	0.673	0.836
Monodepth2-3Ds	Morning	0.277	10.086	41.467	0.290	0.697	0.883	0.950
	Noon	0.290	11.167	46.460	0.315	0.656	0.865	0.936
	Afternoon	0.311	11.901	43.584	0.322	0.618	0.859	0.942
	Night	0.351	14.853	52.417	0.384	0.507	0.790	0.906
	Rain	0.479	26.123	67.677	0.535	0.349	0.621	0.793

To further explore the impact of lighting conditions in data on the depth estimation model, we apply the models with random initial weights and train them on five datasets index of MNv1.3 (see Fig. 10) under different lighting conditions: morning, noon, afternoon, night and rainy day. Quantified results on *AbsRel* are shown in Fig. 11. We can observe that in the lighting conditions at morning and noon, three test models achieve similar results. However, as the lighting in training data is getting dim (afternoon, night, rainy), three models are deteriorated significantly. This can be attributed primarily to that the adequate lighting makes the color between pixels more diverse, and the error map is close to the uniform distribution. Note that at the time of *afternoon*, the models performance dropped dramatically, even worse than *night* that with dimmer lighting condition, we suspect that the reason behind this is there are too much problematic pixels in captured images caused by lens flare, which strongest in *afternoon* compared with the other lighting conditions. SHPH results on the collected sequences under different lighting conditions and different camera moving paths are shown in the row3 of Fig. 10. It can be clearly seen that the clear lighting conditions bring the even distribution of the SHPH.

#### Impact of motion blur

**Figure 12:**
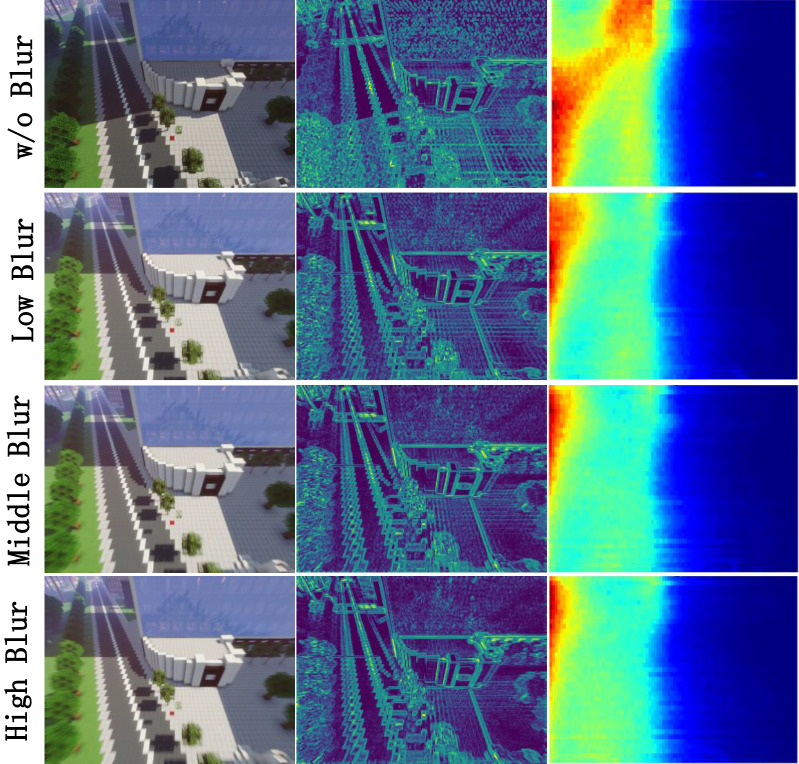
At close to the ground ($\le 70$ m) a histogram statistical result of the inter-frame error and its sequence photometric error heat map.

**Table 6:** The model shows the different accuracy results under different motion blurs.

Train sets (*AbsRel* $\backslash \delta ^1$)	Test datasets			
	*None*	*Low blur*	*Middle blur*	*High blur*
*None*	0.221$\backslash$ 0.731	0.232$\backslash$0.705	0.235$\backslash$0.703	0.237$\backslash$0.704
*Low blur*	0.237$\backslash$0.676	0.203$\backslash$0.731	0.199$\backslash$ 0.748	0.201$\backslash$0.752
*Middle blur*	0.203$\backslash$0.746	0.177$\backslash$0.782	**0.174**$\backslash$ **0.811**	0.179$\backslash$0.808
*High blur*	0.253$\backslash$0.646	0.213$\backslash$0.692	0.197$\backslash$0.729	0.191$\backslash$ 0.754

The best result in each row is underlined and the optimal result is bolded.

The motions of cameras will also affect the stability of the SHPH. As shown in the Fig. 12, it can be seen that the distribution of the photometric error map gradually even with the increase motion blur. In our experiment, four datasets with different motion blur are built. The quantified results of monodepth2 are shown in the Table 6. The motion blur has a great impact on the SHPH, we suspect that it is an effective way to overcome the noise and introduce the robustness by adding a certain motion blur in sequences. This is reflected in SHPH that appropriate motion blur can make the SHPH more stable, which leads the view synthesis of depth estimation model easier (see Fig. 12).

**Table 7:** Motion blur test in monodepth2-3D (up) and monodepth2-3Ds (down). The best result in each row is underlined and the optimal result is bolded.

Train sets (*AbsRel* $\backslash \delta ^1$)	Test datasets			
	*None*	*Low blur*	*Middle blur*	*High blur*
*None*	0.199$\backslash$ 0.768	0.216$\backslash$0.760	0.217$\backslash$0.758	0.218$\backslash$0.752
*Low blur*	0.200$\backslash$0.730	0.185$\backslash$0.764	0.184$\backslash$ 0.765	0.186$\backslash$0.764
*Middle blur*	0.194$\backslash$0.734	0.177$\backslash$0.775	**0.175**$\backslash$ **0.781**	0.176$\backslash$0.785
*High blur*	0.200$\backslash$0.730	0.186$\backslash$0.763	0.180$\backslash$ 0.774	0.181$\backslash$0.778

Train sets (*AbsRel* $\backslash \delta ^1$)	Test datasets			
	*None*	*Low blur*	*Middle blur*	*High blur*
*None*	0.219$\backslash$ 0.739	0.231$\backslash$0.738	0.235$\backslash$0.737	0.233$\backslash$0.735
*Low blur*	0.223$\backslash$0.704	0.207$\backslash$0.734	0.206$\backslash$0.736	**0.205**$\backslash$ **0.737**
*Middle blur*	0.228$\backslash$0.691	0.208$\backslash$0.726	0.207$\backslash$0.731	0.207$\backslash$ 0.734
*High blur*	0.246$\backslash$0.669	0.226$\backslash$0.704	0.224$\backslash$0.706	0.223$\backslash$ 0.707

Table 7 shows the the performance of two variants of monodepth2 in MineNavi datasets with different motion blur. It can be seen that the two models are trained on the motion-blurred dataset, and the performance is significantly better than the dataset without blurred.

Besides, we also introduce vary lighting conditions into experiments. As shown in the Table 5, it can be seen that in the variant models of monodepth2, the darker the lighting conditions, the worse the performance, which is consistent with the results of the experiments. Note that at the time of *afternoon*, the models performance dropped dramatically, even worse than *night* that with dimmer lighting condition, We suspect that the reason behind this is there are too much problematic pixels in captured images caused by lens flare, which strongest in *afternoon* compared with the other lighting conditions (Supplementary Information).

#### Impact of ego-motion variance

The ego-motion of the camera in the video will affect the depth estimation model training. Due to the continuous nature of the camera ego-motion, it is not easy to explore the impact of this factor. In this section, we build three datasets, i.e., MNv2.0, MNv2.1 and MNv2.2, which various in motion mode which corresponds to linear motion $\lambda _1=1$ , overhead cruising motion $\lambda _2 = \frac{\sqrt{2}}{2}$, and circular motion $\lambda _3 =0$ . Finally, the motion speed can be controlled by the number of interval frames of each train triplet in the datasets, and each of them is equipped with three velocities *v*, and $v_3 \>v_2 \>v_1$. We test different motion modes through the models, and the quantitative results are shown in the Table 8. It can be seen that as the $\lambda$ decreases, the performance of the test model also decreases, and the velocity of training triplets also has significantly affect on the performance of test model. According to the previous analysis, the reason behind this probably is that the training triplet with larger $\lambda$ and appropriate velocity have a even distribution in SHPH, hence a better performance is achieved.

**Table 8:** Model performance in different ego-motion modes.

Train$\backslash$test sets (AbsRel $\backslash \delta ^1$)	Velocities		
	$v_1$	$v_2$	$v_3$
$\lambda _1 = 1$	0.143$\backslash$ 0.816	**0.141**$\backslash$ **0.818**	0.152$\backslash$ 0.806
$\lambda _2 = \frac{\sqrt{2}}{2}$	0.240$\backslash$0.472	0.240$\backslash$0.476	0.252$\backslash$0.458
$\lambda _3 = 0$	0.280$\backslash$0.414	0.473$\backslash$0.234	0.481$\backslash$0.248

The best result in each column is underlined and the optimal result is bolded.

#### Velocity of training sequence

**Figure 13:**
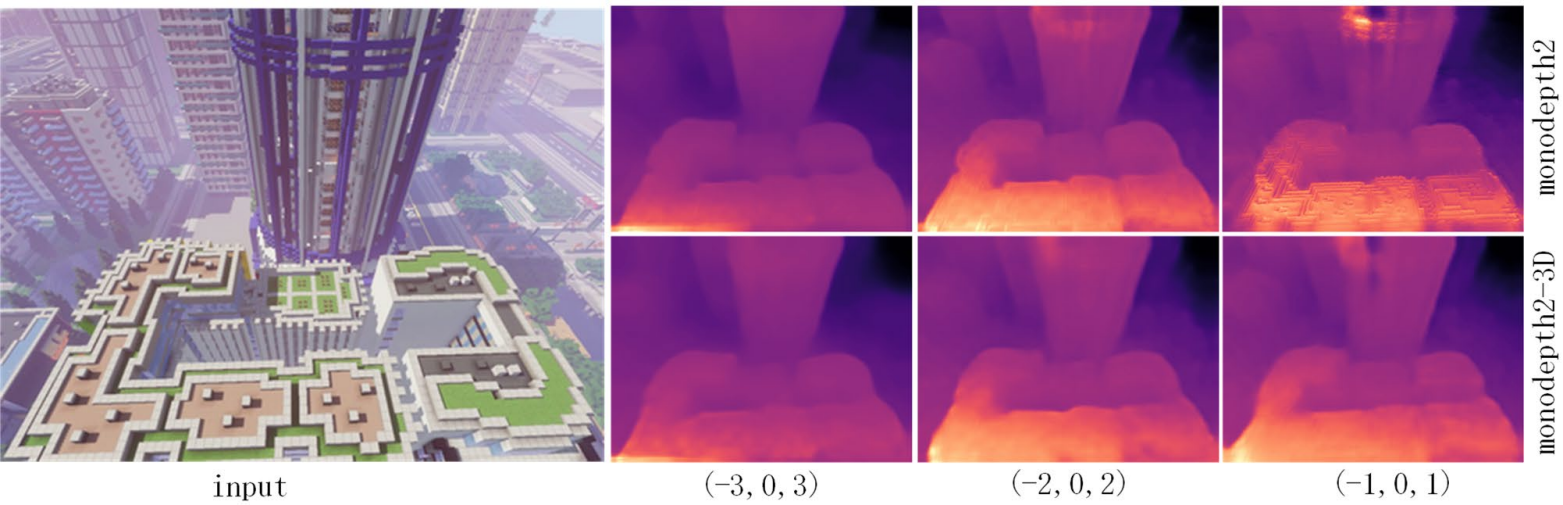
Qualitative model results in MineNavi. First row: monodepth2. Second row: monodepth2-3D. From left to right, the velocity of training sequence is getting lower.

We find that the training sequence vary in sample frequency can greatly affect the performance of the model. It is essential because if the velocity of sampling camera is faster, the photometric differences between two adjacent frames are bigger, making the model difficult to train. Figure 13 shows the qualitative results of the models that vary in velocity of training sequence and encoder.

## Discussion

This paper proposes a method to construct a synthetic dataset, which includes a large-scale scene with low cost but infinite volume, including surface normals, depth, and the 6 DoF paths of the camera’s ego-motion. This dataset generation method can provide a solution to overcome the difficulty of data collection in some dense estimation tasks. For depth estimation task in aircraft navigation, we construct several datasets. According to the experimental results, our proposed dataset generation method can perform as an intermediate domain for depth estimation. The data-to-model experiments reveal that future work should not only focus on the innovation of the models, but also pay more attention to the factors in the dataset that affect the models.

## Supplementary Information

Supplementary Information.

## Data Availability

All MineNavi code for generated dataset used in this analysis can be found at: https://github.com/xdr940/MineNavi. The datasets generated and analysed during the current study are available in the https://www.kaggle.com/datasets/xdr940/minenavi.
