# Supplementary material for: What makes the unsupervised monocular depth estimation (UMDE) model training better
Source: Sci Rep. 2022 Dec 20;12:21999. doi: 10.1038/s41598-022-26613-0 (PMC9768171; doi:10.1038/s41598-022-26613-0)
Supplement: Supplementary file 1 — Supplementary Information. [file 41598_2022_26613_MOESM1_ESM.pdf]

Appendices

A Methods

In this section, we mainly talk about the principle of MDE models, a analysis method of ego-motion video for MDEs and the relevant dataset for the training and evaluation of MDEs in our experiments.

A.1 Deep Models for Monocular Depth Estimation

In order to reconstruct 3D geometry from the ego-motion video by self-supervised method, the main supervision signal comes from the view synthesis between consecutive frames. We aim to infer the intermediate variable, e.g. predicted depth as precisely as possible through this approach. This is an ill-posed problem because a single 2D image may be produced from a large number of distinct 3D scenes, thus it can only be resolved using prior knowledge about the appearance and motion of image sequences.

Assume that I_t and $I_s(I_s \in \{I_{t-1}, I_{t+1}\})$ are two consecutive frames from an unlabeled video, we want to establish the dense pixel correspondence between the two frames. Let p_t denotes the 2D homogeneous coordinate of a pixel in frame I_t and Kdenotes the intrinsic camera matrix. We can compute the corresponding point of p_t in frameI_s using the following equation,

$$p_s = K\hat{T}_{t\to s}\hat{D}_{p_t}K^{-1}p_t \tag{1}$$

where $\hat{D}_t$ is the predicted depth map, and $T_{t\to s}$ is the relative camera pose. We can get the synthesized image $\hat{I}_s$, $I_s = I_t(p_s)$ Thus, the photometric error can be formulated as:

$$L_p = \sum_{s\in\{t^-,t^+\}}\sum_{p}\rho(I_s(p),\hat{I}_s(p)) \tag{2}$$

where $\rho(\cdot)$ is a function to measure the difference of pixels value in the two images. We use a combination of *SSIM*[1] and L1 term as our photometric error function which is formulated as:

$$\rho(I,\hat{I}) = \alpha\frac{1-SSIM(I,\hat{I})}{2} + (1-\alpha)\|I-\hat{I}\| \tag{3}$$

It is robust to illumination changes in a real-world scenario and we set $\alpha = 0.85$. In general, the photometric loss $L_p = \rho(I,\hat{I})$.

However, the supervision signal just based on view synthesis is not informative enough in low-texture or homogeneous regions of the scene due to its ambiguities. Thus, additional regularization is required to learn reasonable depth prediction. A common strategy proposed by Zhou and Godard[2,3], and it is formulated as :

$$L_s = \sum_{p_t}\sum_{d\in x,y}\|\nabla_d^2\hat{D}(p_t)\|_1 e^{-\alpha|\nabla_d I_t(p_t)|} \tag{4}$$

where ∇ is the first derivative along the spatial direction. L_s ensures that the edges in depth map are guided by the images.And overall objective function can be formulated as follows:

$$L^l_{final} = L^l_p + \lambda L^l_s \tag{5}$$

where l stands for multi-scale approach.

A.2 The Sequential Heat-map of Photometric-error Histogram (SHPH)

The unsupervised monocular depth estimation model reconstructs the depth of the scene based on the principle of view synthesis[4,5], i.e. sfmlearner[2], which offer the main supervisory signal during the training. The quality of the view synthesis seriously affects the performance of the model. Thus, we propose a Sequential Heat-map of Photometric-error Histogram (SHPH) to visualize a image sequence (See Figure 1) and verify whether it is compatible with depth estimation model training intuitively.

We have found that during the training, the depth estimation models are very sensitive to the distribution of SHPH, The reason behind is that if the photometric error value in the images is evenly distributed, the model can perform more stable during gradient back-propagation, thereby improving the training efficiency. Accordingly, the histogram of each photometric error map in the training sequence should be kept as even as possible, which also provides a basis for choosing a suitable training sequence.

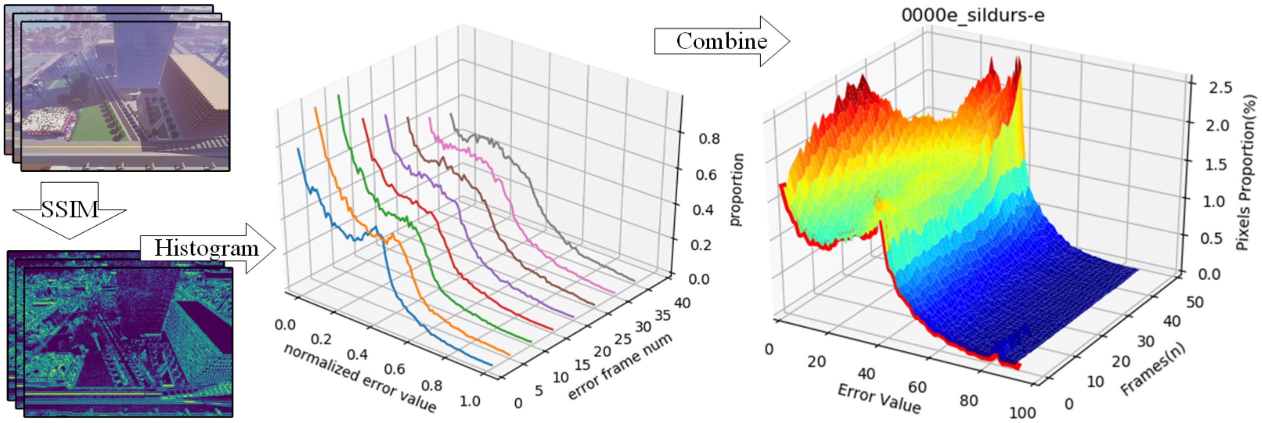

Figure 1. The process of SHPH. In an image sequence, by normalizing the histogram statistics of any adjacent frame photometric error map, and then connecting together, the heat map that reflect the suitableness of training set to MDE could be obtained.

A.3 Datasets

The depth estimation method that based on SfM is not suitable for some datasets due to the absent of successive image[6,7], large scene scale[8] or applicable camera motion[9–11]. Therefore in our experiments, we use the follow datasets to make comparison and evaluate the feasibility of the dataset generation method.

- **KITTI** is widely used in monocular depth learning. In this study, we use KITTI Zhou split[2] that contains image triplets, which static frames with an average optical flow of less than 1 pixel are removed. In our experiment 40k triplets are used for training and 4k triplets for testing.
- **FPV.** To test the performance of depth estimation models in the large-sale scenes, we selected the video data of the first person view (FPV) of the aircraft as another experimental dataset. These dataset cover most of the field scenes, including mountains, woods, lakes, snow mountains and cities. Different with previous UAV datasets[9,10] which captured by simple or even static camera motion, this dataset has diverse moving pattern that suitable for depth estimation model based on SfM. The samples of *fpv* are shown in Figure 2 and we divide all the data into two datasets with the train set volume is 36k and 4k validation set.
- **MineNavi** datasets contain data samples according to different shaders(middle-sildurs, high-sildurs), lighting conditions (morning, moon, evening, night, sunny, and rainy), camera motions(linear motion and circular motion) and image qualities (clear, low blur, middle blur, high blur). We set the field of views of camera as 70 degree. The resolutions of the captured images are 1024×768. The matched depth map ranges from $0.1m$ to $576m$. Finally, we set more than 400 paths around a virtual scene of more than 16 square kilometers, and collected a total of more than 50,000 image sequences. The image scenes include various weather and lighting conditions, with matching depth values.

A.4 Evaluation Metrics

For depth estimation, we evaluate depth estimation models using three accuracy metrics (δ^1, δ^2, δ^3) and four error metrics : absolute relative difference (AbsRel), square related difference (SeqRel), RMSE, log RMSE. Note that the accuracy metrics and *AbsRel* are scale-independent, i.e., the value does not change with the depth range of the dataset. And the others are scale-invariants, i.e., the results evaluated on the dataset with different depth ranges are in different order of magnitudes.

B Related Work

B.1 Synthetic Datasets

Using synthetic data has a long history of application in computer vision community[12–16], it also is a significant data augmentation strategy for data driven models. On one hand, synthesis datasets solve the privacy issues involved in the data collection process partially. On the anther hand, the low-level dense prediction tasks, such as optical flow or depth estimation[17,18], manual annotation is not feasible or even impossible, and synthetic datasets may be the only choice. It is a

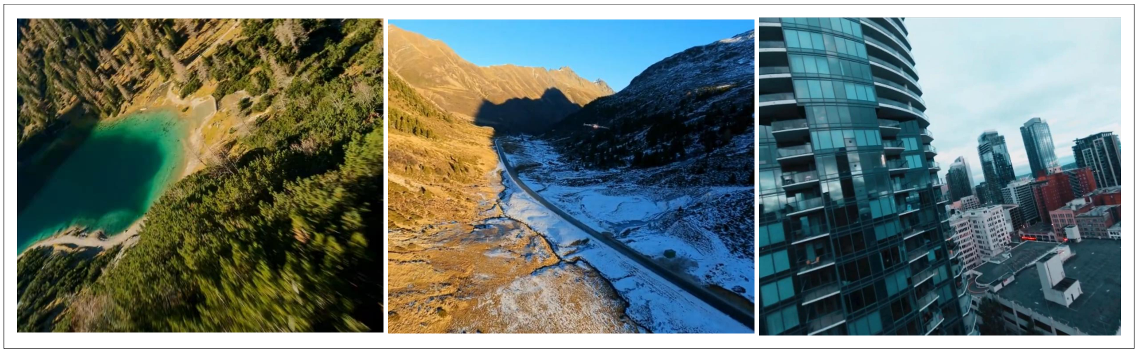

Figure 2. FPV datasets. Left two: Fpv-filed. Right: Fpv-downtown. We introduce FPV dataset to test the generalization of proposed MineNavi dataset.

better idea to use existing scenes from an open-source 3D movie to render artificial annotated data for visual task[17,18]. Using existing game scenes as data source is an ideal alternative[19–22], which decrease the cost of manual 3D modeling of scene and realize capturing the data in arbitrary perspectives. Building datasets through open source games can openly and freely allow users to perform differentiated processing for specific vision task and generate the datasets they need, and through rich plug-ins, the cost and difficulty of building data sets can be further reduced to a certain extent. The related datasets are show in Table 1, we compare MineNavi with EuRoC, KITTI, NYUdepth and other datasets commonly used in SLAM, autonomous driving domain, and conclude that only MineNavi can support depth estimation in scenarios with unlimited amount of data, large scale and diversity.

Table 1. Relevant datasets."V&R" indicates whether the dataset was captured in the virtual or real world, "modal" indicates the data modalities contained in the dataset, where "I" represents the image, "D" represents the depth, "F" represents the optical flow map, and 'N' represents the surface normal mapping of the scene. 'Size' represents the number of RGB image in datasets. 'Img' and 'Seq' indicate that the data is saved as a single image or a sequence of consecutive images, respectively.

					features	
Datasets	V&R	Data Modal	Size	Img&Seq	Resolutions	Scene
NYUDepth[23]	R	I,D	2×10^5	Img	400x600	indoor scene
EuRoC[11]	R	I,D	2×10^5	Img	1024x1024,512x512	indoor,factory scene
MPI-SinTel[18]	V	I,D,F	1×10^4	Seq	1024x436	Specific animation scenes
KITTI[24]	R	I,D,F	4×10^5	Seq	1242x375	outdoor scene
vKITTI[13]	V	I,D,F	2×10^5	Seq	1242x375	outdoor scene.resolutions
**MineNavi(Ours)**	V	I,D,N	∞	Seq	800x600 or higher	unlimited scene(wild,indoor,outdoor)

B.2 Bridging the Reality Gap

Models trained purely on synthetic data often suffer limited generalization caused by domain gap between two type of datasets. Therefore, the utilization and theoretical analysis of the synthetic data set are also very necessary[14]. Domain Randomization (DR)[25] is one of the most promising approaches to make straight-forward transfer learning from synthetic data to real world data.[26] introduced structured domain randomization(SDR) to detection task, which imposes structure onto domain randomization (DR) in order to provide context, they have also shown that pre-training on SDR improves results from real data. Amlan[27] represents the composition of a 3D scene with a scene graph and a probabilistic scene grammar, a common representation in computer graphics. Present works[28] have also devoted significant study to domain adaptation(DA), e.g., the problem of adapting models trained on source domain to a previously unseen target domain. In our proposed MineNavi dataset, we implement DR through multiple lighting conditions rendering, switch shader in the same scene and scene replacement of the same path etc. Due to there is a little overlap between ImageNet and the dataset applied in large-scale depth estimation, MineNavi dataset can be ruled as a intermediate domain for multi-step domain adaptation.

B.3 Datasets Strategy on MDE Training

Although there is a domain gap between synthetic data and real data, most work is based on an assumption that the model trained on real data will perform some results that can be reproduced by training on synthetic data, which leads to the use of

synthetic data as a proxy to explore the influence of factors in real data on the model. Sasiadek et al.[13] analyzed the multi-target detection task on different data with different illumination caused by weather conditions. Su et al.[29] analyzed the influence of background texture and lighting conditions on the task of detecting key points of the object, and concludes that more realistic lighting conditions are beneficial to the performance of the neural network in the task. Nikolaus et al.[30] detailed analysis of the virtual dataset in the optical flow estimation task not only on the impact of lighting, but also included the shape movement and texture of the object. While analysis on above datasets are fairly sufficient, what limits their performance is the lack of the the diversity among the benchmark datasets. To this end, Hu et al.[31] makes a synthetic dataset based on CMU Visual Localization dataset[32] and conducts extensive experimental evaluation on the proposed dataset with several learning-based algorithms. Lasinger et al.[33] introduced a strategy that leverage the mixed datasets im multiple scene for MDE's training. Collecting Large-scale datasets from the Internet[34] is also a way to increase the data diversity, but it require a huge manual labor for data pre- and post-processing.

In this article, we analyze the depth estimation task of weather and lighting conditions, and focus on the influence of camera self-motion, texture and imaging quality on unsupervised monocular depth estimation. To the best of our knowledge, some factors that controlling data source has not been explored before in this context.

References

1. Wang, Z., Bovik, A. C., Sheikh, H. R. & Simoncelli, E. P. Image quality assessment: from error visibility to structural similarity. *IEEE transactions on image processing* **13**, 600–612 (2004).

2. Zhou, T. & Brown, M. Unsupervised learning of depth and ego-motion from video. In *CVPR* (2017).

3. Godard, C. & Aodha, M. Unsupervised monocular depth estimation with left-right consistency. In *CVPR* (2017).

4. Garg, R., Bg, V. K., Carneiro, G. & Reid, I. Geometry to the rescue. In *European conference on computer vision*, 740–756 (Springer, 2016).

5. Flynn, J., Neulander, I., Philbin, J. & Snavely, N. Deepstereo: Learning to predict new views from the world's imagery. In *Proceedings of the IEEE conference on computer vision and pattern recognition*, 5515–5524 (2016).

6. Li, Z. & Snavely, N. Megadepth: Learning single-view depth prediction from internet photos. In *Proceedings of the IEEE Conference on Computer Vision and Pattern Recognition*, 2041–2050 (2018).

7. Nathan Silberman, P. K., Derek Hoiem & Fergus, R. Indoor segmentation and support inference from rgbd images. In *ECCV* (2012).

8. Sturm, J., Engelhard, N., Endres, F., Burgard, W. & Cremers, D. A benchmark for the evaluation of rgb-d slam systems. In *Proc. of the International Conference on Intelligent Robot Systems (IROS)* (2012).

9. Zhu, P., Wen, L., Bian, X., Ling, H. & Hu, Q. Vision meets drones: A challenge. *arXiv preprint arXiv:1804.07437* (2018).

10. Barekatain, M. *et al.* Okutama-action: An aerial view video dataset for concurrent human action detection. In *Proceedings of the IEEE conference on computer vision and pattern recognition workshops*, 28–35 (2017).

11. Burri, M. *et al.* The euroc micro aerial vehicle datasets. *I. J. Robotics Res.* 1157–1163 (2016).

12. Ros, G., Sellart, L., Materzynska, J., Vazquez, D. & Lopez, A. M. The synthia dataset: A large collection of synthetic images for semantic segmentation of urban scenes. In *Proceedings of the IEEE conference on computer vision and pattern recognition*, 3234–3243 (2016).

13. Gaidon, A., Wang, Q., Cabon, Y. & Vig, E. Virtualworlds as proxy for multi-object tracking analysis. In *2016 IEEE Conference on Computer Vision and Pattern Recognition (CVPR)* (2016).

14. Tremblay, J. *et al.* Training deep networks with synthetic data: Bridging the reality gap by domain randomization. In *Proceedings of the IEEE Conference on Computer Vision and Pattern Recognition Workshops*, 969–977 (2018).

15. Guerra, W., Tal, E., Murali, V., Ryou, G. & Karaman, S. Flightgoggles: Photorealistic sensor simulation for perception-driven robotics using photogrammetry and virtual reality. In *2019 IEEE/RSJ International Conference on Intelligent Robots and Systems (IROS)*, 6941–6948 (IEEE, 2019).

16. Antonini, A., Guerra, W., Murali, V., Sayre-McCord, T. & Karaman, S. The blackbird dataset: A large-scale dataset for uav perception in aggressive flight. In *International Symposium on Experimental Robotics*, 130–139 (Springer, 2018).

17. Mayer, N. *et al.* A large dataset to train convolutional networks for disparity, optical flow, and scene flow estimation. In *Proceedings of the IEEE conference on computer vision and pattern recognition*, 4040–4048 (2016).

18. Butler, D. J., Wulff, J., Stanley, G. B. & Black, M. J. A naturalistic open source movie for optical flow evaluation. In *European Conf. on Computer Vision (ECCV)* (2012).

19. Wang, Q., Gao, J., Lin, W. & Yuan, Y. Learning from synthetic data for crowd counting in the wild. In *Proceedings of the IEEE/CVF Conference on Computer Vision and Pattern Recognition*, 8198–8207 (2019).

20. Richter, S. R., Vineet, V., Roth, S. & Koltun, V. Playing for data: Ground truth from computer games. In *European conference on computer vision*, 102–118 (Springer, 2016).

21. Guss, W. H. *et al.* Minerl: A large-scale dataset of minecraft demonstrations. In *IJCAI* (2019).

22. Antonini, A., Guerra, W., Murali, V., Sayre-McCord, T. & Karaman, S. The blackbird dataset: A large-scale dataset for uav perception in aggressive flight. *CoRR* (2018).

23. Eigen. Depth map prediction from a single image using a multi-scale deep network. In *NIPS* (2014).

24. Geiger, A., Lenz, P., Stiller, C. & Urtasun, R. Vision meets robotics: The kitti dataset. *The Int. J. Robotics Res.* **32**, 1231–1237 (2013).

25. Tobin, J. *et al.* Domain randomization for transferring deep neural networks from simulation to the real world. In *2017 IEEE/RSJ international conference on intelligent robots and systems (IROS)*, 23–30 (IEEE, 2017).

26. Prakash, A. *et al.* Structured domain randomization: Bridging the reality gap by context-aware synthetic data. In *2019 International Conference on Robotics and Automation (ICRA)*, 7249–7255 (IEEE, 2019).

27. Kar, A. *et al.* Meta-sim: Learning to generate synthetic datasets. In *Proceedings of the IEEE/CVF International Conference on Computer Vision*, 4551–4560 (2019).

28. Wang, M. & Deng, W. Deep visual domain adaptation: A survey. *Neurocomputing* **312**, 135–153 (2018).

29. Su, H., Qi, C. R., Li, Y. & Guibas, L. J. Render for cnn: Viewpoint estimation in images using cnns trained with rendered 3d model views. In *Proceedings of the IEEE International Conference on Computer Vision*, 2686–2694 (2015).

30. Mayer, N. *et al.* What makes good synthetic training data for learning disparity and optical flow estimation? *Int. J. Comput. Vis.* **126**, 942–960 (2018).

31. Hu, H., Yang, B., Qiao, Z., Zhao, D. & Wang, H. Seasondepth: Cross-season monocular depth prediction dataset and benchmark under multiple environments. *arXiv preprint arXiv:2011.04408* (2020).

32. Badino, H., Huber, D. & Kanade, T. Visual topometric localization. In *2011 IEEE Intelligent vehicles symposium (IV)*, 794–799 (IEEE, 2011).

33. Lasinger, K., Ranftl, R., Schindler, K. & Koltun, V. Towards robust monocular depth estimation: Mixing datasets for zero-shot cross-dataset transfer. *arXiv preprint arXiv:1907.01341* (2019).

34. Li, Z. *et al.* Learning the depths of moving people by watching frozen people. In *Proceedings of the IEEE/CVF Conference on Computer Vision and Pattern Recognition*, 4521–4530 (2019).
